# How does susceptibility to proactive interference relate to speech recognition in aided and unaided conditions?

**DOI:** 10.3389/fpsyg.2015.01017

**Published:** 2015-08-03

**Authors:** Rachel J. Ellis, Jerker Rönnberg

**Affiliations:** Department of Behavioural Sciences and Learning, Linnaeus Centre HEAD, Swedish Institute for Disability Research, Linköping University, Linköping, Sweden

**Keywords:** cognition, speech-in-noise recognition, proactive interference, working memory, executive function, sensorineural hearing loss, hearing aids, older adults

## Abstract

Proactive interference (PI) is the capacity to resist interference to the acquisition of new memories from information stored in the long-term memory. Previous research has shown that PI correlates significantly with the speech-in-noise recognition scores of younger adults with normal hearing. In this study, we report the results of an experiment designed to investigate the extent to which tests of visual PI relate to the speech-in-noise recognition scores of older adults with hearing loss, in aided and unaided conditions. The results suggest that measures of PI correlate significantly with speech-in-noise recognition only in the unaided condition. Furthermore the relation between PI and speech-in-noise recognition differs to that observed in younger listeners without hearing loss. The findings suggest that the relation between PI tests and the speech-in-noise recognition scores of older adults with hearing loss relates to capability of the test to index cognitive flexibility.

## Introduction

Proactive interference (PI) refers to an effect whereby the acquisition of new memories is disrupted by interference from similar information that has been learned previously. PI is a robust phenomenon, having been observed in a variety of contexts including memory for odors (Lawless and Engen, 1977) and the probability of developing post-traumatic stress disorder (Verwoerd et al., 2009). However, PI is traditionally investigated in terms of its effects on memory for semantically-related lists of words (see for example: Wickens et al., 1963; Floden et al., 2000; Ellis and Rönnberg, 2014). The earliest studies of PI focussed only on investigating the capacity to resist PI by presenting lists of words to be recalled after a short interval of time. This procedure is known as the Brown–Peterson paradigm (Brown, 1958; Peterson and Peterson, 1959) and has since been modified to also allow for the examination of release from PI. This modified version of the Brown–Peterson task (Wickens et al., 1963; Wickens, 1970) is based on manipulating the semantic categories of the to-be-remembered word lists such that the first three lists belong to the same category (for example, countries) with the final list belonging to a different one (for example, flowers). Using this paradigm, resistance to PI would be operationalised as the difference in performance (that is, number of words correctly recalled) between the three lists belonging to the same semantic category, with a decrease in performance indicating an effect of PI. The magnitude of release from PI is calculated as the benefit in performance afforded by the change of semantic category between lists three and four.

Effects of PI have been demonstrated in both long-term and short-term memories (Keppel and Underwood, 1962). However, it is the relation between PI and working memory (WM) that is of particular relevance to this study. WM is comprised of both processing and storage components, as opposed to the long-term and short-term memories which simply store information. Thus, rather than simply indexing memory, tests of WM span are thought to measure a number of complex cognitive processes (Sörqvist et al., 2010) including PI (Kane and Engle, 2000; Whitney et al., 2001; Friedman and Miyake, 2004). Studies have also shown that manipulating the degree of PI in tests of WM span affects how well the WM span scores predict performance in other complex cognitive tasks such as tests of prose recall (Lustig et al., 2001) and fluid intelligence (Blalock and McCabe, 2011).

Another complex task, known to be predicted by WM span scores is the perception of distorted speech (see Akeroyd, 2008, for a review). Recent research suggests that performance in a test of PI is significantly related to the speech-in-noise recognition scores of young listeners with normal hearing (Ellis and Rönnberg, 2014). This begs the question of whether the same relation can be observed in older listeners with a hearing loss. According to the ease of language understanding (ELU) model (Rönnberg, 2003; Rönnberg et al., 2008, 2013), when listening conditions are favorable, speech stimuli are implicitly processed, however, if listening conditions are compromised in some way, a mismatch may occur between the stimuli being presented and the representation stored in the long term memory. A mismatch may be caused by many factors, including noise, hearing loss and hearing aid processing and means that explicit processing and storage resources are required, making speech perception more demanding for the listener (Rudner and Rönnberg, 2008). Evidence of increased cognitive load associated with speech perception relative to those with normal hearing has also been observed in cochlear implant users (see for example, Song et al., 2015). Thus, it is expected that a stronger relation between PI and speech-in-noise recognition will be observed in a sample of older listeners with a hearing loss compared to younger listeners with normal hearing. This is due to the fact that the degree of signal distortion, and thus of cognitive resources required to correctly perceive speech-in-noise, is assumed to be greater for older listeners with hearing loss than for younger listeners with normal hearing.

The aim of the present study is therefore to investigate whether the speech-in-noise recognition scores of listeners with an age-related hearing loss is significantly related to performance in a visual PI test. Whether this relation differs depending on whether the speech-in-noise task is completed in an aided or unaided condition will also be investigated, along with the degree to which performance in the PI test relates to aided benefit to speech-in-noise perception.

## Materials and Methods

### Participants

**FIGURE 1:**
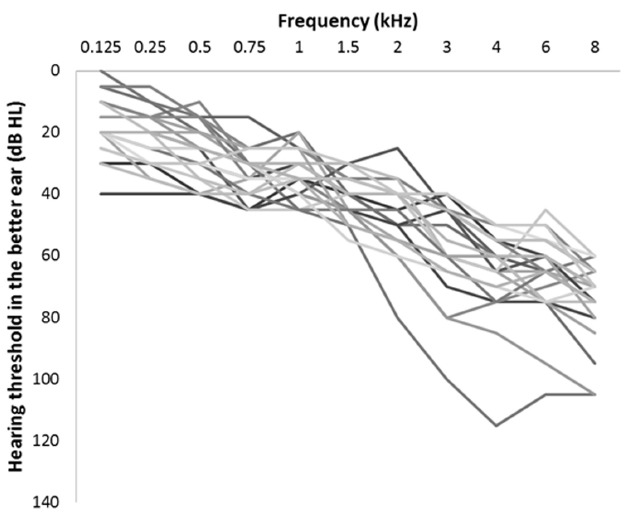
**Hearing thresholds in the better ear for each participant**.

A sample of 23 participants (16 male) aged between 65 and 77 years old (mean age = 70 years) were recruited via the audiology clinic at Linköping University Hospital to take part in the study. Listeners were required to be native speakers of Swedish and have a moderate—to—severe (in two cases, profound at the high-frequencies) symmetrical sensorineural hearing loss and at least 1 year of hearing aid experience, which was binaural for all participants except one. Participants’ better-ear audiograms are displayed in Figure 1. Note that, in two cases, thresholds for some of the high-frequency tones exceeded the maximum presentation level of the audiometer. Where this occurred, the maximum presentation level is recorded as the threshold. The study was approved by the Regional Ethics Board in Linköping (Project code: IBL-2013-00208). Participants were paid 500 SEK for taking part in the study.

### Procedure

All testing was completed in one session, lasting approximately 1.5 h. Upon arrival, participants completed a questionnaire about their hearing loss and a pure-tone audiogram was obtained (at frequencies between 125 and 8000 Hz). Participants then completed the PI test and finally, the speech-in-noise recognition test. The order in which these tests were completed was not counterbalanced, as it was expected that fatigue could affect performance in either of these tests, thus we wished to keep the order the same for all participants. In order to reduce the potential effects of fatigue, participants were encouraged to take breaks in between the tests.

#### Speech-in-noise Recognition Test

Six blocks of 10 sentences from the Swedish HINT corpus (Hällgren et al., 2006) were presented at 65 dB SPL via a loudspeaker situated approximately one meter away from the listener at 0 degrees azimuth. Three of the blocks were presented in an aided condition (using the participant’s own hearing aids) and three in an unaided condition. Allocation of each block to the aided or unaided condition was randomized, as was the order in which the conditions were completed. The sentences were presented in a background of 2-talker babble noise at fixed SNRs between +15 and –3 dB increasing in difficulty in 3 dB steps, similar to the method recommended by Wilson et al. (2007). The first three sentences in each block were presented in quiet so as to minimize the threat of floor effects and to help to maintain participants’ interest in the task. The participants were asked to listen to one sentence at a time and verbally repeat what they heard back to the experimenter. The outcome measure was the mean percentage of keywords correctly identified. The test took approximately 10–15 min to complete.

#### Proactive Interference Test

**FIGURE 2:**
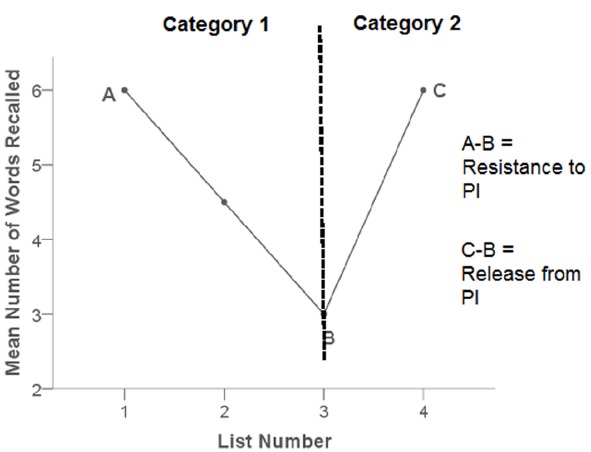
**Diagram showing a typical pattern of PI responses**.

The PI test consisted of three blocks of trials. Each block consisted of four lists of seven words, the first three lists belonging to one semantic category (for example, “capital cities”) and the final list belonging to a second semantic category (for example, “birds”). Words were presented orthographically on a computer screen. After the presentation of each list, participants completed a distractor task for 16 s to prevent rehearsal. The distractor task involved participants being presented (orthographically) with a letter-number sequence (for example, “S56”) and being asked to continue the sequence (“S57, S58, S59” etc). After the distractor task, participants were given 20 s to recall as many words as possible from the list. Participants gave their answers verbally and their responses were noted down by the experimenter. Two outcome measures were then calculated: Resistance to PI (list 1 recall–list 3 recall), where a lower score indicates greater resistance to PI and Release from PI (list 4 recall–list 3 recall), where a higher score indicates greater release from PI. See Figure 2 for a depiction of a typical pattern of PI responses.

Prior to analysis, the normality of the data was confirmed, thus parametric tests were conducted. In order to determine whether there was evidence of an effect PI in the data, *t*-tests were used. Correlational analyses were then conducted to investigate the relation between the measures of speech recognition and those of PI. Partial correlations, with the effect of high frequency pure tone average (HFPTA = average hearing threshold across both ears at 2000, 4000, 6000, and 8000 Hz) removed were also conducted to examine the extent to which the relation between the measures of PI and speech recognition was influenced by degree of hearing loss. Reported p-values are based on 1-tailed hypotheses.

## Results

### Proactive Interference

**FIGURE 3:**
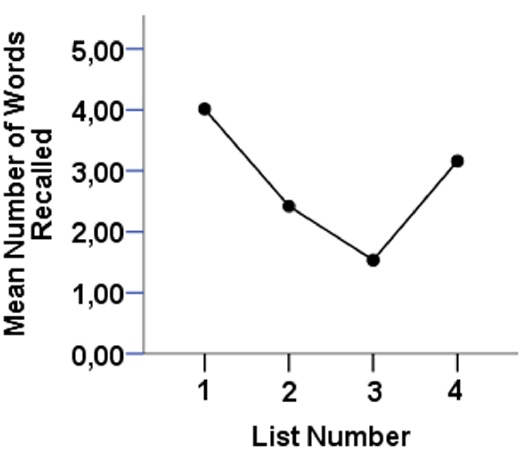
**Pattern of recall in the proactive interference test**.

The mean number of items in each list correctly recalled in the PI task is depicted in Figure 3. The results show that performance steadily declines between lists 1 and 3, then increases again at list 4, a pattern consistent with an effect of PI. Paired-samples *t*-tests confirm significant effects of both resistance to PI [t(68) = 12.34, *p* < 0.000] and release from PI [t(68) = 8.42, *p* < 0.000] thus replicating the expected effects using this task.

### Relation Between PI and Speech-in-noise Recognition

#### SIN Recognition: Unaided

**FIGURE 4:**
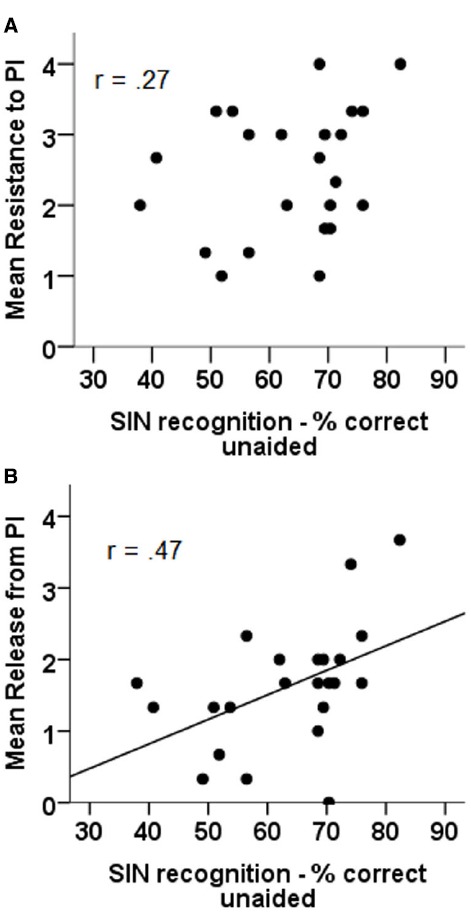
**Correlations between unaided sentence in noise recognition and resistance to PI (A) and release from PI (B)**.

The relation between unaided performance in the SIN test and both resistance to (panel A) and release from PI (panel B) can be seen in Figure 4. The results of correlational analyses indicate that only the relation between unaided SIN performance and release from PI is significant (*r* = 0.47, *p* = 0.015), with the relation between unaided SIN performance and resistance to PI failing to reach significance (*r* = 0.27, *p* = n.s.).

Partial correlational analyses, with the effect of HFPTA removed, revealed the same pattern of results with the relation between unaided SIN and release from PI (*r* = 0.46, *p* = 0.015) showing a significant correlation and the relation between unaided SIN and resistance to PI failing to reach significance (*r* = 0.26, *p* = n.s.).

#### SIN Recognition: Aided

**FIGURE 5:**
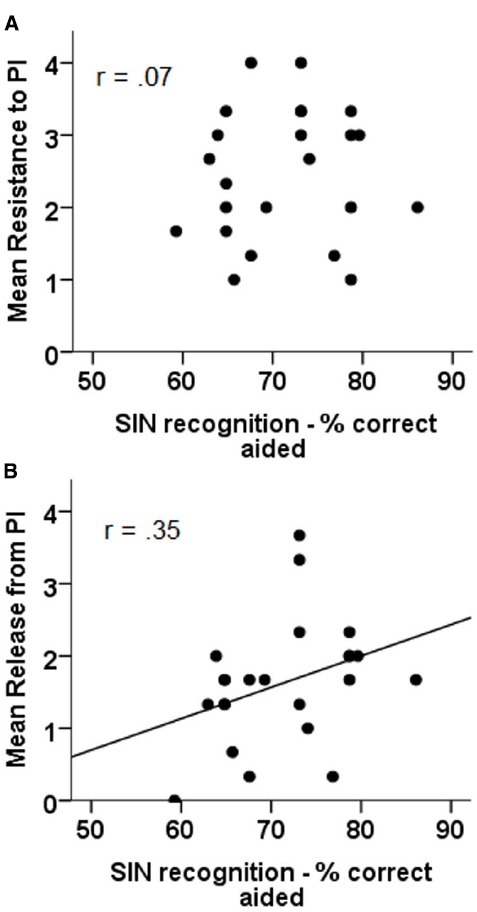
**Correlations between aided sentence in noise recognition and resistance to PI (A) and release from PI (B)**.

The relation between aided performance in the SIN test and both resistance to (panel A) and release from PI (panel B) can be seen in Figure 5. The results of correlational analyses indicate that only the relation between aided SIN performance and release from PI is significant (*r* = 0.35, *p* = 0.05), with the relation between aided SIN performance and resistance to PI failing to reach significance (*r* = 0.07, *p* = n.s.).

Once the effect of HFPTA had been removed, the results of the partial correlational analyses indicated that neither the relation between aided SIN performance and release from PI (*r* = 0.30, *p* = n.s.) nor the relation between aided SIN performance and resistance to PI (*r* = –0.19, *p* = n.s.) were significant.

#### SIN recognition: Aided benefit

**FIGURE 6:**
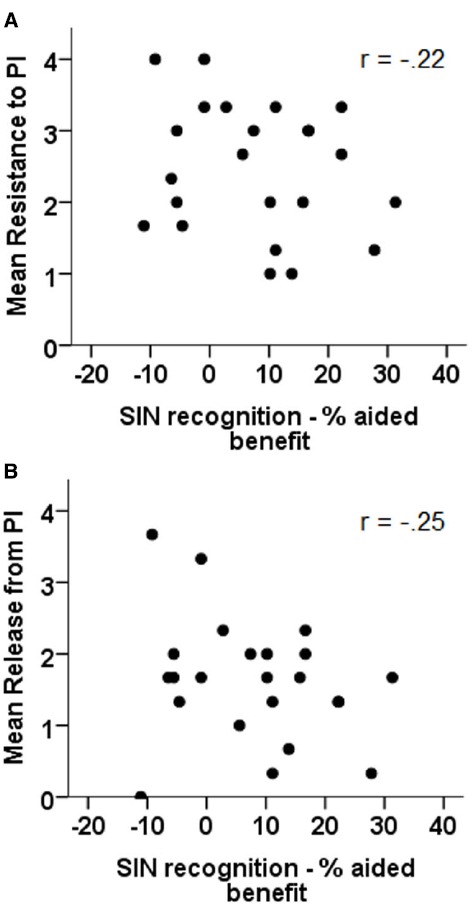
**Correlations between aided benefit in the sentence in noise recognition test and resistance to PI (A) and release from PI (B)**.

The relation between aided benefit in the SIN test and both resistance to (panel A) and release from PI (panel B) can be seen in Figure 6. The results of correlational analyses indicate that neither the relation between aided benefit in the SIN test and release from PI (*r* = –0.25, *p* = n.s.) nor the relation between aided benefit in the SIN test and resistance to PI (*r* = –0.22, *p* = n.s.) were significant.

Partial correlational analyses, with the effect of HFPTA removed, revealed the same pattern of results with neither the relation between aided benefit in the SIN test and release from PI (*r* = –0.25, *p* = n.s.) nor the relation between aided benefit in the SIN test and resistance to PI (*r* = –0.22, *p* = n.s.) reaching significance.

## Discussion

The results of the study provide clear evidence of resistance to and release from PI on a semantically-based word recall task, based on the modified Brown–Peterson paradigm (Brown, 1958; Peterson and Peterson, 1959). The findings indicate that release from PI is significantly correlated with both aided and unaided speech-in-noise recognition in older listeners with hearing loss. Furthermore, the relation between PI and unaided speech recognition continues to be significant even when the effects of loss of high-frequency hearing sensitivity are removed. However, performance on the PI task did not correlate significantly with the degree of benefit to speech-in-noise recognition provided by the use of hearing aids.

### Evidence of Proactive Interference

The results of the study provide evidence of significant effects of both resistance to and release from PI. The magnitude of this effect was greater than that observed in our earlier study on younger listeners with normal hearing (Ellis and Rönnberg, 2014). This difference is likely due to the difference in age of the participant groups with older participants being more affected by interference than younger listeners (see for example, Pettigrew and Martin, 2014). In addition, it is plausible that the nature of the distractor task may have put the older participants at a disadvantage compared to the younger participants as older participants have more difficulty completing tasks involving task switching (see for example, Lawo et al., 2012).

It may also be that, despite the fact that the test of PI used in this case contained no auditory information, that listeners with a hearing loss were disadvantaged anyway, due to the association between hearing loss and cognitive decline (see for example,Rönnberg et al., 2011, 2014). However, as we did not include a control group of older listeners without hearing loss it is difficult to determine whether this is in fact the case.

Due to differences in the methodologies employed, it is difficult to draw direct comparisons between the magnitude of the effects of PI observed in the present study and the results reported in previous studies. However, the only methodological difference between this study and that reported by Ellis and Rönnberg (2014) is that stimuli were presented orthographically rather than aurally as was the case in the earlier study. Thus, it may be that had listeners in our previous study been given the orthographic version of the test, they would have been affected by PI to a greater degree than that observed.

### Proactive Interference and Speech in Noise Recognition

The results of the study indicate that, in the case of older listeners with hearing loss, release from PI correlates significantly with both aided and unaided speech in noise. This pattern of results differs to that observed in younger adults without hearing loss, for whom resistance to, rather than release from, PI was significantly related to speech-in-noise recognition. Furthermore, the magnitude of the observed effects of both resistance to, and release from, PI were greater in the present study than in our earlier study on young adults with normal hearing (Ellis and Rönnberg, 2014).

One possible explanation for these results may relate to the fact that older adults are known to have a greater bias to respond in a context-congruent manner and be less able than younger adults to constrain responses to a given category (Rogers et al., 2012). These tendencies may contribute both to the larger PI effects observed in this older group, and to the difference in how the effects of PI relate to speech-in-noise recognition. We suggest that in both younger and older adults, resistance to PI provides a measure of the capacity to inhibit interference or to direct attention to specific stimuli, capacities which are sufficient to correlate significantly with how well a younger person is able to recognize speech in noise. However, in the case of older adults with hearing loss, we hypothesize that this capacity alone is not sufficient to predict speech in noise recognition, due to fact that speech recognition is more cognitively taxing for this group than for younger adults. Thus we suggest that, in older adults, release from PI may provide an index of the ability to deviate from context, in essence a measure of cognitive flexibility. If this is the case, we would expect release from PI to correlate more strongly with speech-in-noise recognition in conditions in which less contextual information is available, and indeed our results suggest that this is the case. Specifically, once the effects of loss of high frequency hearing sensitivity had been partialled out, release from PI continued to correlate significantly with unaided speech perception, however, ceased to correlate significantly with aided speech recognition. It should be noted that we have made no attempt to disentangle the effects of aging and hearing loss in our data, thus our findings reflect the combined influence of both factors. However, recent research suggests that even when older listeners have normal audiometric thresholds, they tend to perform more poorly on speech perception tests than do younger participants (Füllgrabe et al., 2014). That being the case, we would hypothesize that PI is likely to correlate with speech in noise perception in older adults without hearing loss, however, further research is necessary to investigate this issue.

The fact that that release from PI correlates with speech in noise perception in the unaided condition only is consistent with the ELU model (Rönnberg, 2003; Rönnberg et al., 2008, 2013) if we assume that hearing aids decrease distortion of the signal and allow for more implicit, relatively cognitively undemanding, processing of speech as opposed to the explicit, more cognitively demanding, processing of unaided speech which may be perceived as distorted and inconsistent with representations stored in the long term memory. However, neither measure of PI correlated significantly with the degree of benefit to speech recognition afforded by hearing aid use. There are a number of methodological reasons that may explain this seemingly inconsistent finding. The first is that we did not check how well the hearing aids matched the participants’ prescription. Furthermore, we were unable to check which signal processing options were active in the participants’ hearing aids. There are a number of studies that have linked cognitive status to the success or lack thereof of a particular signal processing strategy to an individual listener (Lunner, 2003; Rudner et al., 2008). Thus it may be that, taken together, these methodological issues may have obscured the relation between PI and aided benefit to speech perception. It may also be of interest to investigate whether the relation between PI and unaided speech perception is affected by regular use of hearing aids, which may affect the degree to which the unaided representations (mis)match with the representations stored in the long-term memory.

The results seem to indicate both that PI is involved in speech perception and that hearing aids facilitate a decreased reliance on cognitive function. The findings seem to be inconsistent with the suggestion that release from PI is an automatic process and unrelated to WM capacity (see Kane and Engle, 2000; Friedman and Miyake, 2004). In the present study, we observe that resistance to and release from PI are significantly correlated with each other indicating that release from PI, at least as measured in the present study, does not simply reflect an automatic process but rather a more explicit process as is the case with resistance to PI. Furthermore, the fact that, after correction for HFPTA, release from PI correlates with speech perception in only the unaided condition, gives further support to the idea that release from PI may be a more complex process that previously thought.

## Author Contributions

RE and JR contributed equally to designing the study, interpreting the data and preparing the manuscript. RE collected the data.

### Conflict of Interest Statement

The authors declare that the research was conducted in the absence of any commercial or financial relationships that could be construed as a potential conflict of interest.

## Abbreviations

HFPTA: High-frequency pure tone average
SIN: Speech in noise
PI: Proactive interference
WM: Working memory.
